# Platelet protease nexin-1 limits fibrinolysis in patients with cirrhosis^☆^

**DOI:** 10.1016/j.jhepr.2025.101563

**Published:** 2025-08-26

**Authors:** Alix Riescher-Tuczkiewicz, Stéphane Loyau, Laurence Venisse, Marion Tanguy, Antoine Wawrzyniak, Emmanuelle de Raucourt, Louise Biquard, Audrey Payancé, Julien Bissonnette, François Durand, Véronique Arocas, Marie-Christine Bouton, Yacine Boulaftali, Pierre-Emmanuel Rautou

**Affiliations:** 1 Université Paris Cité, Inserm, Centre de Recherche sur l'inflammation, UMR 1149, Paris, France; 2 AP-HP, Hôpital Beaujon, Service d'Hépatologie, DMU DIGEST, Centre de Référence des Maladies Vasculaires du Foie, FILFOIE, ERN RARE-LIVER, Clichy, France; 3 INSERM, UMR_S 1148-Laboratory for Vascular Translational Science, Université Paris Cité, Paris, France; 4 Service d’hématologie Biologique, Hôpital Beaujon, Clichy, France

**Keywords:** SerpinE2, Hemostasis, Coagulation, Liver disease, Thrombosis, Thrombin generation assay, Rotational thromboelastometry

## Abstract

**Background & Aims:**

The role of protease nexin 1 (PN-1), a serine protease inhibitor that regulates both coagulation and fibrinolysis, is unknown in patients with cirrhosis. This study aimed to provide a comprehensive assessment of the role of PN-1 in cirrhosis.

**Methods:**

Plasma PN-1 concentration in 212 patients with advanced chronic liver disease and 30 healthy individuals was measured by ELISA. The role of PN-1 was investigated using thrombin generation assay, rotational thromboelastometry, and clot lysis assay in platelet-rich plasma and platelet-free plasma from 10 patients with stable decompensated cirrhosis and 10 healthy individuals.

**Results:**

Plasma PN-1 concentration was higher in patients with advanced chronic liver disease than in healthy individuals (0.21 *vs.* 0 ng/ml, *p* = 0.0001), strongly increased with liver disease severity and portal hypertension, and was associated with 1-year mortality. In patients with stable decompensated cirrhosis, the synergy between platelet PN-1 and thrombomodulin to inhibit thrombin generation, observed in healthy individuals, was lost. Importantly, platelet PN-1 inhibition induced a much greater acceleration of fibrinolysis in patients with cirrhosis than in healthy individuals (1.6 and 2.8 times greater reduction in lysis onset time and lysis index 30, respectively, without tPA). Unlike platelet PN-1, plasma PN-1 plays a negligible role in coagulation and fibrinolysis.

**Conclusions:**

Platelet-derived PN-1 was found to markedly limit fibrinolysis in decompensated cirrhosis, although its impact on coagulation appeared minimal. Although elevated plasma PN-1 concentrations were observed in patients with advanced chronic liver disease, they did not contribute to hemostasis regulation. Overall, PN-1 should be added to the list of hemostasis factors that are dysregulated in cirrhosis.

**Impact and implications:**

The role of PN-1, a serpin that regulates both coagulation and fibrinolysis, is unknown in cirrhosis. PN-1 appears as an important regulator of fibrinolysis in patients with cirrhosis. The role of PN-1 (and platelets more broadly) should be taken into account when assessing fibrinolysis in patients with cirrhosis.

## Highlights

• Platelet PN-1 limits fibrinolysis in cirrhosis but has minimal impact on coagulation.
• Plasma PN-1 is increased in cirrhosis, but it does not regulate haemostasis.
• PN-1 should be added to the list of haemostasis factors dysregulated in cirrhosis.

## Graphical abstract

**Figure:**
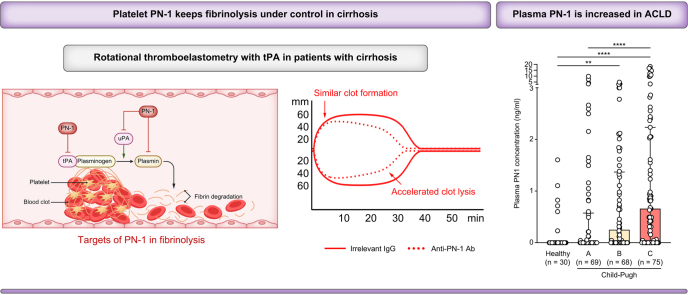

## Introduction

The liver plays a central role in hemostasis, producing most of the proteins involved in coagulation and fibrinolysis.^1^ Consequently, cirrhosis is associated with complex abnormalities in all three phases of hemostasis (*i.e*. primary hemostasis, coagulation, and fibrinolysis), leading to a precarious hemostatic balance.^2^^,^^3^

Serine protease inhibitors (serpin) are proteins that play a key role in the regulation of hemostasis.^4^ Although the role of several serpins, including antithrombin and plasminogen activator inhibitor 1 (PAI-1), has been well described in cirrhosis, the role of protease nexin 1 (PN-1) is unknown.^5^^,^^6^ However, PN-1 has emerged as an important regulator of hemostasis in the field of vascular biology.^7^^,^^8^ PN-1 is a serpin encoded by the SerpinE2 gene and is mainly expressed by platelets, endothelial cells, smooth muscle cells, and fibroblasts.^8^ PN-1 is a potent endogenous inhibitor of thrombin, as well as factor Xa, factor Xia, and activated protein C.^8^^,^^9^ Moreover, PN-1 strongly influences fibrinolysis by inhibiting tissue plasminogen activator (tPA), urokinase plasminogen activator, and plasmin.^10^

The objective of this study was to investigate the role of PN-1 in advanced chronic liver disease (i) by measuring plasma PN-1 concentration and its prognostic value in patients with advanced chronic liver disease and (ii) by evaluating the role of PN-1 in hemostasis in cirrhosis using complementary global hemostasis assays.

## Materials and methods

### Study designs and patients

All the cohorts of patients included in the study are summarized in Table S1.

‘Outcome cohort’: We measured PN-1 concentration in platelet-free plasma (PFP) from 212 patients from the MICROSPY cohort. This cohort prospectively included patients with advanced chronic liver disease (METAVIR F3/F4) who underwent hepatic vein and/or right heart catheterization at Beaujon Hospital (Clichy, France) between June 2013 and June 2016.^11^ Clinical, laboratory, and hemodynamic features were prospectively collected on the day of catheterization, and PFP was prepared on the same day. Diagnosis of advanced chronic liver disease was based either on histological criteria or on the combination of clinical, laboratory, morphological, and hemodynamic features. Non-inclusion criteria were as follows: history of transjugular intrahepatic portosystemic shunt or liver transplantation, extrahepatic malignancy, presence of portal vein thrombosis, anticoagulant and/or antiplatelet therapy within 10 days before inclusion, hepatocellular carcinoma outside the Milan criteria, HIV infection, primary sclerosing cholangitis and primary biliary cholangitis, Budd–Chiari syndrome, or an acute event (hepatorenal syndrome, bacterial infection, alcoholic hepatitis, and variceal bleeding) within 2 weeks before inclusion. Of the 212 patients, 91 have been included in a previous study.^11^ We also included 30 healthy individuals as controls.

‘PN-1 liver cohort’: We included prospectively 24 patients with cirrhosis (all severity stages) who had blood drawn from the hepatic vein and superior vena cava during hepatic catheterism. In both sites, blood was drawn through an MPA catheter (HNB7-0-38-100-P–NS–MPA; Cook, Ireland). We measured the PN-1 concentration in the plasma from the hepatic vein and superior vena cava.

‘TGA and ROTEM cohorts’: We assessed the role of PN-1 in hemostasis in 10 patients with stable decompensated Child–Pugh B or C cirrhosis and 10 healthy individuals (same inclusion and exclusion criteria as the outcome cohort). We performed in these patients (i) a thrombin generation assay (TGA) on platelet-rich plasma (PRP) and PFP, (ii) a rotational thromboelastometry (ROTEM®) on PRP, and (iii) a measurement of plasma tPA and PAI-1 concentration.

‘Clot lysis assay cohort’: To assess the role of plasma PN-1 in fibrinolysis, a clot lysis assay was performed on PFP from 10 patients in the MICROSPY cohort with known elevated plasma PN-1 concentration (see Supplementary methods).^11^

‘PN-1 and PAI-1 platelet cohort’: We included seven patients with stable decompensated cirrhosis and seven healthy individuals (same inclusion and exclusion criteria as the outcome cohort). We measured PN-1 and PAI-1 concentrations in the supernatant of washed platelets following activation with thrombin receptor-activating peptide-6 (TRAP-6), to assess PN-1 release upon platelet activation, and after thermal lysis using liquid nitrogen, to evaluate the total intracellular pool of PN-1.

### Sample preparation and measurement

#### Preparation of plasma and washed platelets, PN-1 and tPA concentration measurement by ELISA, hepatic venous pressure gradient measurement, and p-selectin and C-reactive protein measurement

Detailed methods are provided in Supplementary methods.[12], [13], [14], [15], [16], [17]

#### Thrombin generation assay

TGA on PRP: TGA was performed using the calibrated automated thrombogram assay (CAT®, Diagnostica Stago, Asnieres, France) according to the manufacturer’s instructions. Briefly, in a 96-well microplate, 80 μl of PRP was mixed with 20 μl of triggering reagent containing tissue factor at 1 pmol/L (PRP Reagent, Stago) with or without thrombomodulin (TM) (9-RABTMM-4202, Cryopep, Montpellier, France). TM was used at the final concentration that inhibits 50% of the endogenous thrombin potential (ETP) of normal plasma. Plasma was pre-incubated for 15 min with an irrelevant antibody (immunoglobulin G [IgG]; 011-000-003, Jackson, Suffolk, UK) or a polyclonal blocking PN-1 antibody (in-house antibody, previously published in Aymonnier *et al.*) at a final concentration of 150 μg/ml.^18^ After incubation for 10 min at 37 °C, 20 μl of a mixture of the fluorogenic substrate (Z-Gly-Gly-Arg-AMC, Stago) (final concentration 417 μmol/L) and calcium chloride (final concentration 16.7 nmol/L) was distributed automatically to the test system. Measurements were performed in triplicate for each plasma every 20 s for 60 min.

TGA on PFP was performed as mentioned above, except for the triggering reagent containing tissue factor at 1 pmol/L and phospholipids at 4 μmol/L (PPP reagent low, Stago).

#### Rotational thromboelastometry

ROTEM was conducted using the four-channel ROTEM® Delta device. Briefly, PRP was pre-incubated with 100 μg/ml irrelevant IgG (011-000-003, Jackson) or polyclonal anti-PN-1 blocking antibody for 15 min. Clotting was initiated by adding 20 μl of extrinsically activated ROTEM assay reagent containing tissue factor (ExTEM, Tem International, Munich, Germany) and 20 μl of buffered concentrated calcium chloride solution (Star-TEM reagent, Tem International) to 300 μl of PRP at 37 °C in a preheated cup. tPA-ROTEM using tPA (Actilyse, Boehringer Ingelheim, Ingelheim, Germany) at a concentration of 75 ng/ml was conducted to assess fibrinolysis. The parameters investigated were as follows: (i) lysis onset time, defined as the time (s) from clotting to the start of significant lysis, indicated by a decrease in amplitude of 15% compared with maximum clot firmness; (ii) lysis index at 30 and 45 min after clotting, corresponding to the ratio of the amplitude to maximum clot firmness at each specified time point (%); (iii) maximum lysis, defined as the maximum lysis detected during the run, calculated as the difference between maximum clot firmness and the lowest amplitude after maximum clot firmness (in % of maximum clot firmness); and (iv) lysis time, defined as the time (s) from clotting until clot firmness decreases to 10% of maximum clot firmness during fibrinolysis.

To minimize variability in the TGA and the ROTEM assay, all experiments were performed by the same operator using the same equipment and timing.

### Statistical analysis

Quantitative variables were expressed as median (IQR) and were compared using the Mann–Whitney *U* test or the Wilcoxon test, as appropriate. Qualitative variables were expressed as absolute and relative (percentage) frequencies and compared using the Chi-square test or Fisher’s test, as appropriate. Spearman’s correlation and stepwise linear regression analyses were conducted to investigate potential associations between plasma PN-1 concentration and clinical, laboratory, or hemodynamic features.

Follow-up time was defined as the time from blood collection to the date of liver transplantation, death, or last follow-up visit. Competitive risk analysis was performed using a multistate model, as recommended for patients with cirrhosis.^19^ Data for patients who had not died were censored at the date of the last follow-up visit and were coded 0; data for patients who died before liver transplantation were coded 1; and liver transplantation was considered to be a competing risk event, and data were coded 2. A cumulative incidence function of death was calculated to describe the probability of death at a given time and was reported at 1 year with a 95% CI. The value of plasma PN-1 concentration with the best sensitivity and specificity in the area under the receiver operating characteristic curve analysis (Youden’s index) for death at 1 year was chosen for further analyses. Plasma PN-1 concentration (continuous variable) and PN-1 Youden’s index (categorical variable) were introduced into a multivariable Fine and Gray proportional hazards model with the model for end-stage liver disease (MELD) score to adjust for the severity of advanced chronic liver disease and determine whether PN-1 had a prognostic value independently of this score.

The power calculation for hemostasis tests (ROTEM, TGA, and clot lysis assay) was based on our experience and validated using statistical methods. We determined a group size of n = 10 to detect a 1.5-fold change with a power of 0.8 and a *p* value <0.05. The level of significance was set at a two-sided *p* value <0.05. All statistical analyses were performed using IBM SPSS Statistics 29 (IBM, New York, NY, USA), R version 3.4.1 (R Foundation for Statistical Computing, Vienna, Austria), or GraphPad Prism 10 (GraphPad Software Inc., San Diego, CA, USA).

### Ethics

The study was conducted in accordance with the principles of the Declaration of Helsinki and was approved by the local ethics committee (IRB 11-112 for the outcome and PN-1 liver cohorts and Comité de protection des personnes Ile de France II, #22.03009.000142, for the other cohorts). All patients and healthy individuals provided written informed consent for study participation.

## Results

### Plasma PN-1 concentration increases in advanced chronic liver disease and with advanced chronic liver disease severity

We first investigated plasma PN-1 concentrations in patients with advanced chronic liver disease and the features associated with these concentrations to gain an insight into the potential sources of PN-1.

A total of 212 patients from the outcome cohort were included. Their characteristics are presented in Table 1. The main cause of advanced chronic liver disease was excessive alcohol consumption. The median Child–Pugh and MELD scores were 8[6], [7], [8] and 14,[9], [10], [11], [12], [13], [14], [15], [16], [17], [18], [19], [20] respectively. Thirty healthy individuals were also included, with a median age of 52 (39-56) years, of whom 22 (73%) were men.

**Table 1:** Patients’ characteristics at blood draw.

	n	Outcome cohort (n = 212)	n	TGA cohort (n = 10)	n	ROTEM cohort (n = 10)
**Clinical features**						
Age (years)	212	56 (50–62)	10	55 (50–62)	10	56 (48–64)
Male sex, n (%)	212	145 (68)	10	9 (90)	10	8 (80)
BMI (kg/m^2^)	212	26 (23–30)	10	24.4 (22–26.3)	10	25.5 (23.7–28.2)
Cardiovascular risk factors, n (%)	212		10		10	
Hypertension		83 (39)		1 (10)		1 (10)
Smoking		67 (32)		5 (50)		4 (40)
Diabetes		62 (29)		1 (10)		1 (10)
Dyslipidemia		18 (9)		0		0
Causes of liver disease ∗	212		10		10	
Excessive alcohol consumption		110 (52)		8 (80)		7 (70)
MASLD		43 (20)		1 (10)		1 (10)
Hepatitis C		52 (25)		1 (10)		1 (10)
Hepatitis B		19 (9)		1 (10)		1 (10)
Other		26 (12)		1 (10)		2 (20)
Ascites^†^	212	114 (54)	10	8 (80)	10	7 (70)
Large varices esophageal or history of band ligation	137	31 (23)	8	6 (75)	8	6 (75)
Hepatocellular carcinoma	212	66 (31)	10	0	10	0
Child–Pugh class	212		10		10	
A		69 (33)		0		0
B		68 (32)		4 (40)		6 (60)
C		75 (35)		6 (60)		4 (40)
MELD	212	14 (9–20)	10	17 (12–21)	10	16 (11–19)

**Laboratory data**						
Serum sodium (mmol/L)	212	136 (134–138)	10	133 (136–138)	10	135 (134–138)
Serum creatinine (μmol/L)	212	70 (61–84)	10	59 (49–66)	10	61 (56–80)
Serum AST (ULN)	212	1.7 (1.2–2.7)	10	56 (41–77)	10	51 (41–64)
Serum ALT (ULN)	211	0.9 (0.5–1.4)	10	23 (21–35)	10	23 (21–31)
Serum bilirubin (μmol/L)	212	34 (15–79)	10	50 (31–131)	10	37 (26–59)
Serum albumin (g/L)	212	32 (25–38)	10	27 (25–31)	10	29 (24–31)
Leukocytes (10^9^/L)	212	5.4 (4.3–7.1)	10	6.1 (4.9–6.8)	10	6 (4.5–6.7)
Hemoglobin (g/dl)	212	12.3 (10.4–13.8)	10	12.6 (9.6–13)	10	12.6 (10–13)
Platelet count (10^9^/L)	212	101 (71–140)	10	94 (59–107)	10	95 (62–123)
C-Reactive protein (mg/L)	186	6 (2–13)	7	5 (2–29)	9	5 (1.5–20.5)
Prothrombin rate (%)	212	57 (44–77)	10	42 (38–57)	10	43 (39–61)

**Hemodynamic data**						
HVPG (mmHg)	200	16 (12–20)	9	18 (17–24)	9	18 (16–24)
Heart rate (bpm)	208	76 (66–88)	9	83 (68–92)	9	83 (66–92)
Mean arterial pressure (mmHg)	208	94 (85–102)	9	92 (76–96)	9	89 (76–96)
Right atrial pressure (mmHg)	203	4 (3–7)	9	4 (3–5)	9	4 (3–5)
Mean pulmonary artery pressure (mmHg)	193	15 (12–19)	9	15 (10–17)	9	15 (10–16)
Cardiac index (L/min/m^2^)	192	3.5 (2.8–4.4)	9	3.9 (3.1–5.7)	9	3.5 (2.7–5.7)
Beta blocker treatment	211	81 (38)	10	2 (20)	10	2 (20)

Data are presented as median (range) or n (%). ∗Patients can have several causes of liver disease associated. ^†^Ascites presence was defined here as the presence of mild/moderate or abundant ascites (referring to 2 or 3 points on the Child–Pugh classification). ALT, alanine aminotransferase; AST, aspartate aminotransferase; bpm, beat per minute; MASLD, metabolic dysfunction-associated steatotic liver disease; MELD, model for end-stage liver disease; ROTEM, rotational thromboelastometry; TGA, thrombin generation assay; ULN, upper limit of normal.

**Fig. 1:**
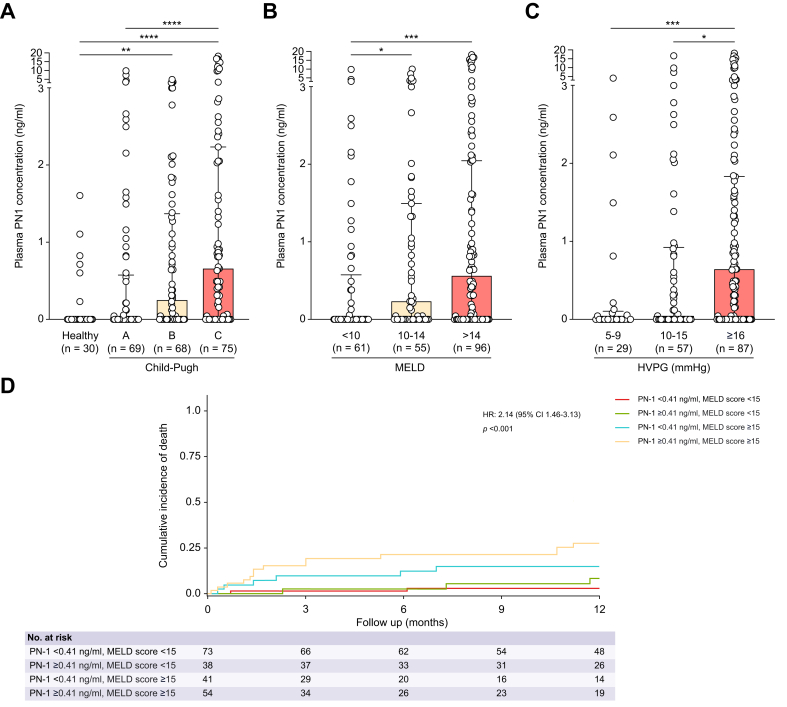
Plasma PN-1 concentration in patients with advanced chronic liver disease according to liver disease severity and its association with mortality at 1 year. (A) Child–Pugh score, (B) MELD score, (C) HVPG, and (D) cumulative incidence of death according to MELD score and plasma PN-1 concentration (follow-up available for 206 patients). The upper end of the box corresponds to the median, and the IQR is represented by horizontal bars. Comparisons were made using the Kruskal–Wallis test, followed by Dunn’s multiple comparisons test. Univariate regression analyses were conducted using the Fine and Gray proportional hazards models. HR, hazard ratio; HVPG, hepatic venous pressure gradient; MELD, model for end-stage liver disease; PN-1, protease nexin 1.

Plasma PN-1 concentration was detected more frequently in patients with advanced chronic liver disease than in healthy individuals (58% *vs.* 20%, *p* = 0.0001). The median plasma PN-1 concentration was 0.21 (0–1.37) ng/ml in patients with advanced chronic liver disease compared with 0 (0–0) ng/ml in healthy individuals (*p* = 0.0001) and significantly increased with liver disease severity (Fig. 1A and B). Plasma PN-1 concentration also increased with portal hypertension severity, namely hepatic venous pressure gradient (HVPG) and ascites (Fig. 1C and Table S2). Features associated with plasma PN-1 concentration, as determined by univariate analysis, are shown in Table S2 and S3. All features reaching a significant *p* value in the univariate analysis were included in a multivariable analysis using a stepwise linear regression approach. Serum bilirubin and C-reactive protein were independently associated with plasma PN-1 concentration (regression coefficient 0.009, 95% CI 0.004–0.014, *p* = 0.0002; and regression coefficient 0.051, 95% CI 0.02–0.082, *p* = 0.001, respectively; n = 172). In contrast, sex, arterial hypertension, diabetes, alcohol-related advanced chronic liver disease, metabolic dysfunction-associated steatotic liver disease (MASLD)-related advanced chronic liver disease, hepatocellular carcinoma, age, serum sodium, serum creatinine, aspartate aminotransferase (AST), serum albumin, hemoglobin, prothrombin rate, HVPG, mean arterial pressure, cardiac index, and MELD score were not associated.

We then compared PN-1 concentrations in plasma samples drawn from hepatic veins and the superior vena cava in 24 patients with advanced chronic liver disease (‘PN-1 liver cohort’; Table S4) and found no significant difference (0 [0–0.94] *vs.* 0 [0–0.83] ng/ml, *p* = 0.94). The same result was obtained when restricting the analysis to patients with Child–Pugh B/C cirrhosis (0 [0–1.70] *vs.* 0.09 [0–1.63] ng/ml, *p* = 0.38, n = 12). Plasma PN-1 concentration correlated with markers of systemic inflammation, namely C-reactive protein (n = 203; r = 0.254, *p* <0.001), but not with markers of platelet and endothelial activation, namely P-selectin (n = 205; r = 0.077, *p* = 0.271) (data not shown). These findings suggest that the increase in plasma PN-1 observed in advanced chronic liver disease is unlikely to originate from the liver, platelets, or endothelium but rather seems linked to systemic inflammation.

During the 1-year follow-up, 64 (30%) patients underwent liver transplantation, and 25 (11%) patients died. Considering liver transplantation as a competing risk, patients with plasma PN-1 concentration above the Youden’s index-optimized cut-off value (*i.e.* PN-1 ≥0.41 ng/ml) had a higher incidence of death compared with those with plasma PN-1 concentration below this threshold (20% [12–29%] *vs.* 7% [3–13%], *p* = 0.014). However, plasma PN-1 concentration (below or above 0.41 ng/ml) was not associated with the incidence of death at 1 year when the analysis was adjusted for the MELD score (adjusted hazard ratio 2.21, 95% CI 0.92–5.30, *p* = 0.077). When patients were categorized using an MELD cut-off value of 15, those with an MELD score of ≥15 and a plasma PN-1 concentration above the Youden’s index-optimized cut-off showed a higher cumulative incidence of death (Fig. 1D).

### PN-1 does not synergize with TM to inhibit thrombin generation in PRP from patients with cirrhosis, unlike in healthy individuals

To better understand the role of PN-1 in coagulation in patients with stable decompensated cirrhosis, we performed TGA using PN-1 blocking antibody or non-immune immunoglobulin, with and without the addition of soluble TM. As platelets are the main source of PN-1 under physiological conditions, we first performed TGA on PRP from patients with cirrhosis and from healthy individuals, with the platelet count of the healthy individuals adjusted to that of the patients with cirrhosis. The characteristics of the patients are presented in Table 1.

**Fig. 2:**
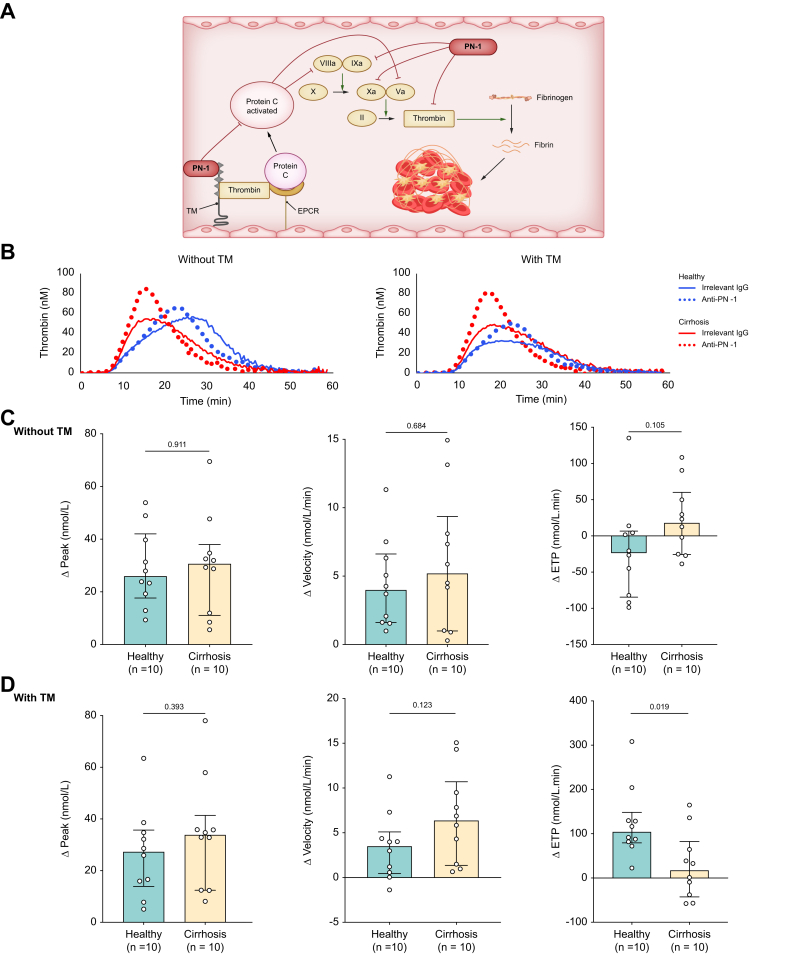
Comparison of the effect of PN-1 inhibition on thrombin generation assay parameters between patients with cirrhosis and healthy individuals on platelet-rich plasma. (A) Illustration of the action of PN-1 on coagulation. (B) Thrombin generation plot in cirrhosis patients and healthy individuals without and with anti-PN-1 antibody/thrombomodulin. Changes in peak, velocity, and ETP values with anti-PN-1 antibody *vs.* irrelevant IgG (C) without and (D) with TM. Comparisons were made using the Mann–Whitney *U* test. ETP, endogenous thrombin potential; IgG, immunoglobulin g; PN-1, protease nexin 1; TM, thrombomodulin.

When comparing patients with cirrhosis and healthy individuals in the presence of an irrelevant antibody, we observed a lower ETP in patients with cirrhosis in the absence of TM (*p* = 0.0029) and a resistance to TM (Table 2). In the absence of TM, the anti-PN-1 blocking antibody had a similar effect in patients with cirrhosis and healthy individuals, namely a change in thrombin generation profile with a higher peak, a higher velocity, and a reduced time to tail, but without any effect on ETP (Table 2, Fig. 2C, and Fig. S1). In the presence of TM, the same results were observed (Table 2, Fig. 2D, and Fig. S1), except for ETP. Although the anti-PN-1 blocking antibody partially suppressed ETP reduction induced by TM in healthy individuals, it had no effect on ETP in patients with cirrhosis. This result may be explained by a tendency for a shorter time to tail in patients with cirrhosis compared with healthy individuals (*p* = 0.0524).

**Table 2:** Results of thrombin generation assay in PRP in healthy individuals and patients with cirrhosis.

PRP	Healthy individual (n = 10)		Patient with cirrhosis (n = 10)	
	Irrelevant IgG	Anti-PN-1	Irrelevant IgG	Anti-PN-1
**Without thrombomodulin**				
Lag time (min)	8.3 (7.4–10.8)	8.9 (8.2–11.4)∗∗	8.9 (6.1–10.3)	9.3 (7.4–10.7)∗∗
Peak (nM)	72.1 (57.3–95.3)	106.8 (79.1–128.1)∗∗	55.4 (46.2–75.5)	83.4 (68–118.7)∗∗
Time to peak (min)	22.2 (19–26.6)	21.2 (18.7–22.5)∗	18.5 (15.7–22.8)	17 (15.6–18.6)^†^
Endogenous thrombin potential (nmol/Lxmin)	1,500 (1,366–1,658)	1,445 (1,309–1,730)	1,029 (861–1,404)^††^	1,069 (898–1,396)^††^
Velocity (nmol/L/min)	5.7 (4.1–7.2)	9.2 (6.5–13.7)∗∗	5.6 (3.9–8)	11.1 (8.1–15)∗∗
Time to tail (min)	54.6 (46.7–59)	44 (40.8–48.6)∗∗	52.9 (48.1–58.5)	44.5 (40.7–47.3)∗∗

**With thrombomodulin**				
Lag time (min)	9.2 (7.9–11.7)	9.6 (8.3–13)∗	10.3 (6.9–11.1)	10.7 (7.6–11.2)∗
Peak (nmol/L)	49.1 (41.5–63.3)	76.3 (53.6–103)∗∗	47.6 (39.3–66.3)	79.9 (67.7–113.1)∗∗
Time to peak (min)	19 (16.3–23.7)	19.7 (16.4–22.6)	20.4 (16.5–23.6)	17.3 (16.5–19.5)
Endogenous thrombin potential (nmol/Lxmin)	874 (784–990)	1,023 (858–1,144)∗∗	967 (760–1,168)	1,003 (810–1,278)
Velocity (nmol/L/min)	5.2 (3.4–7.6)	9.3 (5.4–12.46)∗	5.1 (3.1–6.7)	11.5 (7.8–15)∗∗
Time to tail (min)	52.8 (45.5–59.6)	44.5 (39.5–47.7)∗∗	54.4 (49.2–62.7)	42.9 (39.4–47.7)∗∗
Endogenous thrombin potential ratio	0.60	0.69∗∗	0.90	0.91

Data are presented as median (range). ∗Statistical significance for the comparisons between the condition with irrelevant IgG and anti-PN-1 antibody (in the group of patients with cirrhosis or in the group of healthy individuals). Comparisons were performed using the Wilcoxon test. ∗*p* <0.05 for anti-PN-1 *vs*. PRP irrelevant IgG, ∗∗*p* <0.01, and ∗∗∗*p* <0.001. ^†^Statistical significance for the comparisons between patients with cirrhosis and healthy individuals (irrelevant IgG in healthy individuals *vs*. irrelevant IgG in patients with cirrhosis, or anti-PN-1 in healthy individuals *vs*. anti-PN-1 in patients with cirrhosis). Comparisons were performed using the Mann–Whitney *U* test. ^†^*p* <0.05 for patients with cirrhosis with irrelevant IgG or anti-PN-1 *vs*. healthy individuals with irrelevant IgG or anti-PN-1, ^††^*p* <0.01, and ^†††^*p* <0.001. IgG, immunoglobulin G; PN-1, protease nexin 1; PRP, platelet-rich plasma.

We then performed TGA on PFP. As expected, patients with cirrhosis and healthy individuals had similar ETP when the test was performed without TM. With TM, patients with cirrhosis had a higher ETP than healthy individuals, likely because of TM resistance, well known in patients with cirrhosis (Table 3). Unlike in PRP, inhibition of PN-1 in PFP had no or mild effect on TGA parameters in patients with cirrhosis and healthy individuals, although some comparisons reached statistical significance (Table 3). Response to the anti-PN-1 antibody was similar in patients with cirrhosis and healthy individuals (Fig. S2).

**Table 3:** Results of thrombin generation assay in PFP in healthy individuals and patients with cirrhosis.

PFP	Healthy individual (n = 10)		Patient with cirrhosis (n = 10)	
	Irrelevant IgG	Anti-PN-1	Irrelevant IgG	Anti-PN-1
**Without thrombomodulin**				
Lag time (min)	5.6 (5–6.3)	5.3 (4.8–6)	4.8 (4.2–5.5)	4.6 (4.2–5.5)∗
Peak (nM)	115 (103–176)	124 (105–162)	137 (101–160)	141 (103–161)
Time to peak (min)	9.7 (9.3–10.8)	9.6 (9.2–10.5)	7.7 (7.1–8.6)^††^	7.4 (6.8–8.5)∗∗^,†^
Endogenous thrombin potential (nM⸱min)	941 (872–1,081)	947 (831–1,058)	916 (766–996)	898 (733–986)∗
Velocity (nM/min)	25.6 (23.5–46.5)	29.7 (22.8–39.3)	48.3 (30–53.6)^†^	53.5 (31.3–56.6)
Time to tail (min)	28.8 (25–30.8)	27.1 (24.5–30.2)	29.6 (27.5–31.8)	28.4 (26.1–30.5)∗∗

**With thrombomodulin**				
Lag time (min)	5.8 (5.3–6.9)	5.5 (5–6.4)∗∗	5.1 (4.5–5.4)^†^	4.8 (4.3–5.5)
Peak (nM)	106 (78–146)	94 (77–138)	135 (98–163)	139 (99–165)
Time to peak (min)	9.2 (8.5–10.5)	8.8 (8.2–9.9)∗∗	7.7 (7.2–8.2)^††^	7.4 (7.1–8.1)^††^
Endogenous thrombin potential (nM⸱min)	560 (401–751)	492 (401–693)	819 (667–964)^†^	807 (675–946)^†^
Velocity (nM/min)	32.8 (22.3–44.7)	30.1 (22.7–42.1)	49 (31.5–60)	55.7 (31.5–67.8)
Time to tail (min)	23.4 (22.2–27.3)	22.7 (21.3–26.6)∗∗	28.1 (25.8–30.5)^†^	27.3 (25.6–29.6)∗∗^,†^

Data are presented as median (range). ∗Statistical significance for the comparisons between the condition with irrelevant IgG and anti-PN-1 antibody (in the group of patients with cirrhosis or in the group of healthy individuals). Comparisons were performed using the Wilcoxon test. ∗*p* <0.05 for PN-1 *vs*. PRP irrelevant IgG, ∗∗*p* <0.01, and ∗∗∗ *p* <0.001. ^†^Statistical significance for the comparisons between patients with cirrhosis and healthy individuals (irrelevant IgG in healthy individuals *vs*. irrelevant IgG in patients with cirrhosis, or anti-PN-1 in healthy individuals *vs*. anti-PN-1 in patients with cirrhosis). Comparisons were performed using the Mann–Whitney *U* test. ^†^*p* <0.05 for patients with cirrhosis with irrelevant IgG or anti-PN-1 *vs*. healthy individuals with irrelevant IgG or anti-PN-1, ^††^*p* <0.01, and ^†††^*p* <0.001. IgG, immunoglobulin G; PN-1, protease nexin 1; PFP, platelet-free plasma.

Altogether, these results suggest that, unlike in healthy individuals, in patients with cirrhosis, platelet PN-1 does not synergize with TM to inhibit thrombin generation. Plasma PN-1 appears to play a negligible role in both patients with cirrhosis and healthy individuals.

### Platelet PN-1 limits early fibrinolysis in patients with cirrhosis, whereas plasma PN-1 does not

We then investigated the role of PN-1 in fibrinolysis in stable decompensated cirrhosis by performing ROTEM in PRP. The characteristics of the patients are presented in Table 1.

**Fig. 3:**
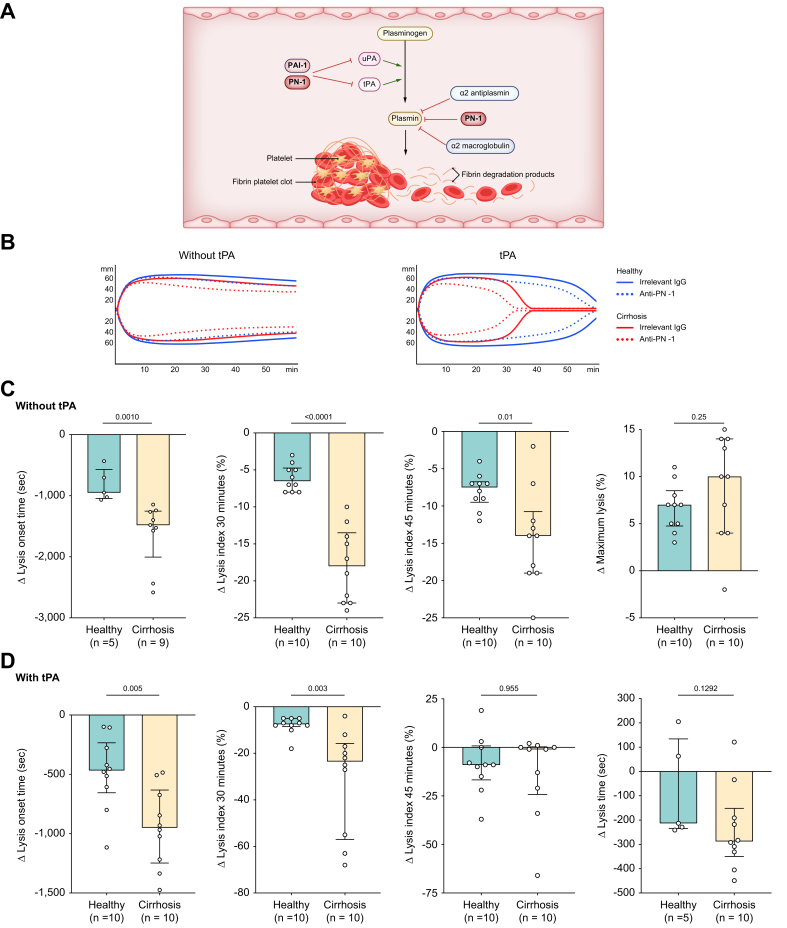
Comparison of the effect of PN-1 inhibition on rotational thromboelastometry parameters between patients with cirrhosis and healthy individuals on platelet-rich plasma. (A) Illustration of the action of PN-1 on fibrinolysis. (B) Rotational thromboelastometry plot in healthy individuals and patients with cirrhosis without and with anti-PN-1 antibody/tPA. Changes in lysis onset time, lysis index at 30 min, lysis index at 45 min, maximum lysis, and lysis time values with anti-PN-1 antibody *vs.* irrelevant IgG (C) without and (D) with tPA. In the condition without tPA, lysis onset time occurred in five healthy individuals and nine patients with cirrhosis. Comparisons were made using the Mann–Whitney *U* test. IgG, immunoglobulin g; PN-1, protease nexin 1; tPA, tissue plasminogen activator.

Without tPA, significant lysis allowing calculation of lysis onset time occurred in five of 10 healthy individuals and nine of 10 patients with cirrhosis. Among these, PN-1 inhibition induced earlier fibrinolysis in patients with cirrhosis than in healthy individuals, as shown by a shorter lysis onset time in the former than in the latter (Table 4), as well as by a 1.6 times greater reduction in lysis onset time induced by PN-1 inhibition in patients with cirrhosis than in healthy individuals (Fig. 3C). In both groups, PN-1 inhibition significantly reduced the lysis index at 30 and 45 min, but the reduction was 2.8 times (lysis index at 30 min) and 1.9 times (lysis index at 45 min) greater in patients with cirrhosis than in healthy individuals (Fig. 3C). PN-1 inhibition increased maximum lysis in both groups, but the effect was not different between them (Fig. 3C).

**Table 4:** Results of rotational thromboelastometry in PRP in healthy individuals and patients with cirrhosis.

PRP	Healthy individual (n = 10)		Patient with cirrhosis (n = 10)	
	Irrelevant IgG	Anti-PN-1	Irrelevant IgG	Anti-PN-1
**Without tPA**				
Lysis onset time (min)∗	3,164 (3,145–3,406)	2,443 (2,151–2,672)	2,497 (2,061–3,081)	835 (722–1,398)^‡‡,§§§^
Lysis index at 30 min (%)	98 (98–98)	92 (90–94)^‡‡^	94 (88–96)^§§§§^	75 (72–79)^‡‡,§§§§^
Lysis index at 45 min (%)	91 (90–92)	84 (82–84)^‡‡^	85 (80–90)^§§^	72 (66–74)^‡‡,§§§§^
Maximum lysis (%)	15 (14–18)	21 (20–25)^‡‡^	25 (18–26)^§§^	31 (28–37)^‡‡,§§§^

**With tPA**				
Lysis onset time (min)	2,627 (2,210–2,855)	2,155 (1,927–2,229)^‡‡^	1,927 (1,691–2,094)^§§^	921 (729–1,172)^‡‡,§§§§^
Lysis index at 30 min (%)	97 (96–98)	90 (88–91)^‡‡^	87 (74–94)^§§§^	69 (12–72)^‡‡,§§§§^
Lysis index at 45 min (%)	82 (34–88)	66 (18–79)	1 (0–47)^§§^	1 (0–5)^§§^
Maximum lysis (%)	95 (43–100)	99 (52–100)	100 (100–100)^§^	100 (100–100)
Lysis time (s)^†^	2,945 (2,477–3,185)	2,779 (2,388–3,150)	2,432 (2,143–2,955)	2,362 (1,815–2,623)^‡‡^

Data are presented as median (range). ∗In the condition without tPA, LOT occurred in 5 healthy individuals and 9 patients with cirrhosis. ^†^In the condition with tPA, LT occurred in five healthy individuals and 10 patients with cirrhosis. ^‡^Statistical significance for the comparisons between the condition with irrelevant IgG and anti-PN-1 antibody (in the group patients with cirrhosis or in the group of healthy individuals). Comparisons were performed using the Wilcoxon test. ^‡^*p* <0.05 for PN-1 *vs*. PRP irrelevant IgG, ^‡‡^*p* <0.01, and ^‡‡‡^*p* <0.001. ^§^Statistical significance for the comparisons between patients with cirrhosis and healthy individuals (irrelevant IgG in healthy individuals *vs*. irrelevant IgG in patients with cirrhosis, or anti-PN-1 in healthy individuals *vs*. anti-PN-1 in patients with cirrhosis). Comparisons were performed using the Mann–Whitney *U* test. ^§^*p* <0.05 for patients with cirrhosis irrelevant IgG or anti-PN-1 *vs*. healthy individuals with irrelevant IgG or anti-PN-1, ^§§^*p* <0.01, ^§§§^*p* <0.001, and ^§§§§^*p* <0.0001. IgG, immunoglobulin G; LT, Lysis time; LOT, Lysis onset time; PN-1, protease nexin 1; PRP, platelet-rich plasma; tPA, tissue plasminogen activator.

With tPA, PN-1 inhibition also induced earlier fibrinolysis in both groups, as indicated by a shorter lysis onset time (Table 4), and the reduction was 2 times greater in patients with cirrhosis than in healthy individuals (Fig. 3D). The lysis index at 30 min was also significantly reduced in both groups, and the reduction was 3.1 times greater in patients with cirrhosis than in healthy individuals with PN-1 inhibition (Fig. 3D). The lysis index at 45 min was not significantly reduced by PN-1 inhibition in the presence of tPA. Maximum lysis was ≥99% in all patients with cirrhosis without the anti-PN-1 antibody, so the addition of the anti-PN-1 antibody had no impact. Sufficient lysis allowing calculation of lysis time occurred in five (50%) healthy individuals and in all patients (100%) with cirrhosis. Lysis time was significantly decreased by the anti-PN-1 antibody in patients with cirrhosis but not in healthy individuals (Table 4).

We also investigated fibrinolysis in PFP using a clot lysis assay. We observed no effect of PN-1 inhibition on clot lysis (Fig. S3), suggesting that platelet PN-1, but not plasma PN-1, plays a role in fibrinolysis in patients with cirrhosis.

### Platelet count and plasma PN-1 expression do not explain the effect on fibrinolysis in cirrhosis

**Fig. 4:**
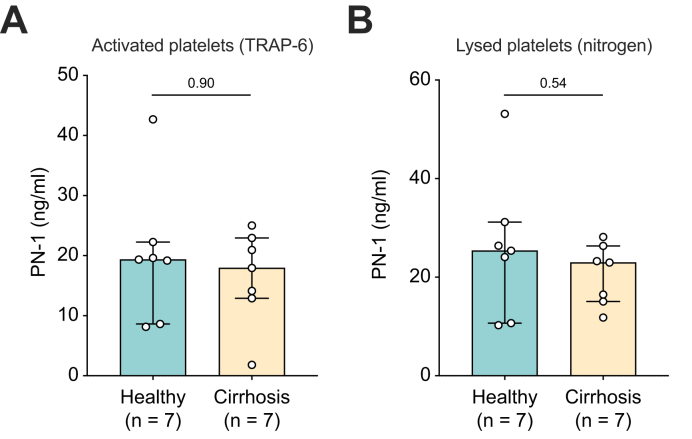
PN-1 concentration in the supernatant after platelet activation (TRAP-6) or platelet lysis (nitrogen). PN-1 concentration in the supernatant of washed platelets after (A) platelet activation (TRAP-6) and (B) platelet lysis (nitrogen) in seven healthy individuals and seven patients with stable decompensated cirrhosis (Child–Pugh B/C). Comparisons were made using the Mann–Whitney *U* test. PN-1, protease nexin 1; TRAP-6, thrombin receptor-activating platelet-6.

To gain insight into the mechanisms by which PN-1 regulates fibrinolysis in cirrhosis, we assessed PN-1 concentration in platelets from seven healthy individuals and from patients with Child–Pugh B/C cirrhosis (Table S4). As shown in Fig. 4, no difference was observed between the two groups either after platelet activation with TRAP-6 or after thermal lysis. We also performed ROTEM in PRP, adjusting the platelet count of five healthy individuals to their own blood counts rather than to those of patients with cirrhosis. The results were consistent with those obtained when platelet counts were adjusted to the levels seen in patients with cirrhosis, further reinforcing the conclusion that platelet count and platelet PN-1 concentration were not responsible for the observed effect on fibrinolysis. The results are shown in Fig. S4, Table S5 and Fig. 4.

We then measured the target of PN-1 in fibrinolysis, namely tPA, and observed a much higher plasma tPA concentration in patients with cirrhosis than in healthy individuals (8 [5.5–9.7] *vs.* 2 [1.1–2.6] ng/ml, *p* <0.0001).

In addition, we measured plasma PAI-1 concentrations in 10 patients with cirrhosis and healthy individuals from the ROTEM cohort. The median PAI-1 concentration was significantly higher in patients with cirrhosis than in healthy individuals (21.5 [11.5–24.5] *vs*. 7.4 [5.3–10.4] ng/ml, *p* = 0.003). However, we found no significant difference in platelet PAI-1 expression between seven healthy individuals and patients with cirrhosis (Child–Pugh B/C), either after platelet activation with TRAP-6 or after thermal lysis (Fig. S5).

## Discussion

PN-1 has emerged as an important regulator of coagulation and fibrinolysis in the past two decades.^7^^,^^8^^,^^10^^,^^18^ Although hemostasis of patients with cirrhosis has been widely studied, no data were available on the role of PN-1 in this setting. This study aimed to fill this gap in knowledge.

The first major finding of this study was that PN-1 is an important regulator of fibrinolysis in patients with stable decompensated cirrhosis. Indeed, when PN-1 activity was inhibited in PRP in ROTEM, fibrinolysis occurred earlier and was more pronounced at 30 and 45 min in patients with decompensated cirrhosis than in healthy individuals. This effect was not explained by higher PN-1 concentrations in platelets of patients with decompensated cirrhosis, as we found similar concentrations to those in healthy individuals, both after TRAP-6 stimulation and platelet lysis. Plasma PN-1 did not appear to play a key role either, as there was no effect of PN-1 inhibition in a clot lysis assay performed on PFP. However, we observed that the main target of PN-1 in fibrinolysis, namely tPA, had increased plasma concentration in patients with decompensated cirrhosis, consistent with the literature.^20^^,^^21^ We can thus hypothesize that PN-1, in decompensated cirrhosis, counteracts the pro-fibrinolytic effect of increased tPA and thus helps keep fibrinolysis ‘under control’. Our results indicate that, even if PAI-1, a major inhibitor of fibrinolysis, has higher concentrations in the plasma of patients with decompensated cirrhosis than in healthy individuals, this increase may not be sufficient to counterbalance the excessive tPA concentrations. As a result, when PN-1 is inhibited, PAI-1 fails to compensate for the elevated tPA activity, leading to enhanced fibrinolysis. In addition, previous work has shown that platelet PN-1 and PAI-1 act synergistically in the regulation of fibrinolysis.^10^ Consistently, our data showed that inhibiting PN-1 did not fully increase fibrinolysis in patients with decompensated cirrhosis, suggesting the involvement of other players. One limitation of our experiments is that we focused solely on platelet and plasma PN-1 by conducting ROTEM in PRP, without considering other cell types that also express PN-1, such as endothelial cells or fibroblasts. These cells may play a significant role in the regulation of fibrinolysis *in vivo*, potentially affecting the overall hemostatic balance that our study did not capture.

The second major finding of this study was that the role of PN-1 in the regulation of coagulation in patients with stable decompensated cirrhosis was not as important as that observed in healthy controls. Indeed, inhibition of PN-1 had no effect on ETP in cirrhosis, but it moderately increased ETP in healthy individuals. The explanation for this finding may lie, on the one hand, in the known resistance of patients with cirrhosis to the action of TM^22^ and, on the other hand, in the inhibitory activity of the PN-1/TM complex on thrombin, which is notably enhanced when the two act together, compared with acting individually.^9^ We can thus hypothesize that the addition of an anti-PN-1 antibody in cirrhosis was simply less effective than in healthy individuals because the resistance to TM led to a less potent synergy of the PN-1/TM complex. Our observation that PN-1 plays a more predominant role in the regulation of fibrinolysis than of coagulation in patients with cirrhosis is unusual, as PN-1 better inhibits thrombin than tPA (Ka = 6–8.3 × 10^5^ M^-1^ s^-1^ for thrombin *vs.* Ka = 1.2–3 × 10^4^ M^-1^ s^-1^ for tPA). The complex coagulation status of patients with cirrhosis might account for this specificity. Another limitation of our study is that we only explored the role of PN-1 in patients with stable decompensated cirrhosis, and its role potentially differs in other contexts known to further impair hemostasis, such as acute decompensation of cirrhosis and acute on chronic liver failure.

The third major finding of this study was that plasma PN-1 concentration was higher in patients with advanced chronic liver disease than in healthy individuals, and this increase correlated with the severity of liver disease. Our results show that the liver is not responsible for this increase in plasma PN-1 in advanced chronic liver disease. As plasma PN-1 concentration was independently associated with serum bilirubin concentration, we can speculate that a reduction in hepatic clearance may be a contributing factor. PN-1 complexes are internalized for degradation by low-density lipoprotein receptor-related protein 1 (LRP1), which is expressed by the liver.^23^^,^^24^ Because plasma PN-1 concentration was also independently associated with serum C-reactive protein concentration, increased production by endothelial cells upon exposure to inflammatory mediators might be another explanation, similar to what is observed with tPA.^20^ These hypotheses warrant further investigation.

## Conclusions

This study demonstrates that PN-1 should be considered as a new player contributing to the altered hemostasis of patients with stable decompensated cirrhosis, playing mostly an antifibrinolytic role, thus keeping fibrinolysis ‘under control’. Plasma concentration of PN-1 strongly increased with advanced chronic liver disease severity, but it did not improve the prediction of mortality over existing scores.

## Abbreviations

CLA, clot lysis assay; ETP, endogenous thrombin potential; HVPG, hepatic venous pressure gradient; IgG, immunoglobulin G; MELD, model for end-stage liver disease; PAI-1, plasminogen activator inhibitor 1; PN-1, protease nexin 1; PFP, platelet-free plasma; PRP, platelet-rich plasma; ROTEM, rotational thromboelastometry; serpin, serine protease inhibitor; TGA, thrombin generation assay; TM, thrombomodulin; tPA, tissue plasminogen activator; TRAP-6, thrombin receptor-activating platelet-6.

## Financial support

This work has been funded by Université Paris Cité (IDEX UP-2021-I-053 AAP EMERGENCE 2021; ANR-18-IDEX-0001) and by Agence Nationale de la Recherche (ANR-25-CE14-4488). AR-T received the ‘poste d’accueil Inserm’ fellowship. P-ER’s research laboratory is supported by the Fondation pour la Recherche Médicale (FRM EQU202303016287), the ‘Institut National de la Santé et de la Recherche Médicale’ (ATIP AVENIR), the ‘Agence Nationale pour la Recherche’ (ANR-18- CE14-0006-01, RHU QUID-NASH, and ANR-22-CE14-0002), ‘Émergence, Ville de Paris’, Fondation ARC, the EU’s Horizon 2020 research and innovation programme under grant agreement no. 847949, and France 2030 RHU LIVER-TRACK. YB is supported by ANR-18-IDEX-0001 from Université Paris Cité and by ANR-22-CE17-0052.

## Authors’ contributions

Conception and design of the study: AR-T, SL, LV, M-CB, YB, P-ER. Generation, collection, assembly, analysis and/or interpretation of data: AR-T, SL, LV, MT, AW, EdR, LB, AP, JB, FD, VA, M-CB, YB, P-ER. Drafting or revision of the manuscript: AR-T, SL, M-CB, YB, P-ER. Approval of the final version of the manuscript: AR-T, SL, LV, MT, AW, EdR, LB, AP, JB, FD, VA, M-CB, YB, P-ER.

## Data availability

The data that support the findings of this study are available from the corresponding author upon reasonable request.

## Conflicts of interest

The authors declare no conflicts of interest that pertain to this work.

Please refer to the accompanying ICMJE disclosure forms for further details.
