# Supplementary material for: Platelet protease nexin-1 limits fibrinolysis in patients with cirrhosis
Source: JHEP Rep. 2025 Aug 26;7(12):101563. doi: 10.1016/j.jhepr.2025.101563 (PMC12639314; doi:10.1016/j.jhepr.2025.101563)
Supplement: Multimedia component 1 [file mmc1.pdf]

Platelet protease nexin-1 limits fibrinolysis in patients with cirrhosis

Alix Riescher-Tuczkiewicz, Stéphane Loyau, Laurence Venisse, Marion Tanguy, Antoine Wawrzyniak, Emmanuelle de Raucourt, Louise Biquard, Audrey Payancé, Julien Bissonnette, François Durand, Véronique Arocas, Marie-Christine Bouton, Yacine Boulaftali, Pierre-Emmanuel Rautou

Table of contents

Supplementary methods……………………………………………………………..……...2

Supplementary tables……………………………………………………………………7

Supplementary figures…………………………………………………………………14

Supplementary methods

Plasma preparation

PFP was prepared according to a standardized protocol proposed by Lacroix and colleagues[12]. Briefly, venous blood was collected with a 21-gauge tourniquet needle in 0.129 mol/L citrated tubes, after having discarded the first milliliter of blood. Tubes then remained motionless in the upright position at room temperature for a maximum of 4 hours until platelet-free plasma preparation, consisting of two successive centrifugations, each of 15 minutes at 2,500 g at 20°C with a light brake. Aliquots of platelet-free plasma were then stored at -80°C until use.

PRP was obtained from venous blood collected in citrated tubes and centrifuged, within maximum 1 hour after sampling, for 15 minutes at 120 g at 20°C. For TGA and ROTEM, platelet count in the PRP of healthy individuals was adjusted to that of the patient with cirrhosis, using autologous PFP.

Preparation of washed platelets

Blood was collected in one-tenth volume ACD-A (38 mmol/L citric acid, 60 mmol/L sodium citrate, 136 mmol/L glucose). Because patients with cirrhosis have thrombocytopenia, we adapted our protocol[13] to maximize platelet recovery. Platelet-rich plasma (PRP) was obtained by centrifugation at 120 g for 15 min. Coagulation in PRP and platelet activation were prevented by the addition of 10 µg/mL apyrase (PY0627100, Agrobio) and 100 nmol/L prostaglandin E1 (Sigma) and 1/10 ACD-A. The PRP was centrifuged at 1200g for 15 min and the platelet pellet was resuspended in washing buffer (pH 6.5, 103 mmol/L NaCl, 5 mmol/L KCl, 1 mmol/L MgCl2, 5 mmol/L glucose, 36 mmol/L citric acid, apyrase 10µg/mL, PGE1 100 nmol/L, human serum albumin 3.5mg/mL). Platelets were centrifuged at 80g for 5 minutes to remove residual

red blood cells. The supernatant was centrifuged again at 1200g for 12 minutes. Platelets were resuspended at 5.10^8/mL in reaction buffer consisting of 5 mmol/L Hepes, 137 mmol/L NaCl, 2 mmol/L KCl, 1 mmol/L MgCl2, 12 mmol/L NaHCO3, 0.3 mmol/L NaH2PO4, 5.5 mmol/L glucose, pH 7.4, containing 3.5 mg/mL BSA. Then 1.10^8 platelets were either activated with TRAP-6 (SFLLRN amide; Bachem, Bubendorf, Switzerland) 50 µmol/L or thermally lysed with successive cycles of nitrogen freezing followed by thawing at 37°C. The platelets were then centrifuged at 10000g and the supernatant was collected for measurement of PN-1 and PAI-1 concentrations.

PN-1 concentration measurement by ELISA

Microlon-Med half-well high binding plates (Greiner bio-one, Courtaboeuf, France) were coated for 72 hours at 4°C, with 0.5 µg of capture antibody per well (MA-57B11)[14]. After washing with phosphate buffered saline with Tween (PBS-T) buffer (PBS, 0.1 %; bovine serum albumin (A7030-100G, Sigma) 0.002 %; Tween 20 (P1379, Sigma)), wells were saturated for 2 hours at room temperature with 100 µL of PBS containing 1% bovine serum albumin. Wells were then washed four times with PBS-T buffer. Calibration was performed using different concentrations (0-50 ng/mL) of recombinant Escherichia coli–expressed human PN-1 (rPN-1) that was produced and purified as previously described and used as antigen[15]. Platelet free plasma or supernatant from washed platelets was incubated in the wells overnight at 4°C. After washing in PBS-T buffer, bound PN-1 was probed with a detection antibody (0.4 µg/mL of MA-55F11-HRP) for 2 hours at room temperature, and detected via hydrolysis of 3,3′,5,5′-tetramethylbenzidine (DY999, Biotechne) during 15 minutes. The reaction was stopped with 25 µl of sulfuric acid (H2SO4 3M). Absorbance was read at 485 nm. We

previously reported that intra-assay and inter-assay coefficients for this ELISA are 6.2% and 11.1%, respectively [14].

tPA concentration measurement by ELISA

tPA was measured in the PFP using the Zymutest tPA Antigen ELISA Kit (RK011A, Hyphen). This assay quantifies both free tPA and tPA complexed to inhibitors, with a sensitivity of 0.5 ng/mL.

Clot lysis assay

To assess the role of plasma PN-1 in fibrinolysis, we performed clot lysis assay on PFP derived from 10 patients with elevated plasma PN-1 concentration (median plasma PN-1 concentration of 3ng/mL (2.1-9.1)). Briefly, clots were formed in a 96-well half area microtiter plate (Greiner Bio-One, Germany) with a total reaction volume of 75 µL per well. In each well, 4 µL of irrelevant IgG (011-000-003, Jackson) or Polyclonal blocking PN-1 antibody at final concentration of 100 µg/mL was incubated 15 minutes at room temperature with 40 µL of PFP from healthy individuals or patients with cirrhosis. Six µL of a 3 ng/mL final concentration of Tissue-type plasminogen activator (t-PA) (Actilyse, Boerhinger Ingelheim, Germany) was added and coagulation initiated by adding 25 µL per well of CaCl2 (12.5 mmol/L). tPA concentration used in this experiment was evaluated *a priori,* by testing several concentrations and choosing the one that was the most appropriate to detect a change in fibrinolysis. Turbidity was measured at 405 nm every 6 minutes at 37°C with plate sealers for 250 cycles of reading. Fibrinolytic parameter investigated was the clot lysis time (CLT) expressed in min corresponding to the time from 50% maximal clotting to 50% lysis.

Hepatic venous pressure gradient measurement

Hepatic venous pressure gradient (HVPG) was assessed using a technique previously described[16, 17]. Briefly, after an overnight fasting, local anesthesia was performed and an introducer was placed under ultrasound guidance using the Seldinger technique. A 7 French balloon catheter was inflated in the right or median hepatic vein and wedged hepatic venous pressure was measured. Then, free hepatic venous pressure was obtained. HVPG was calculated as the difference between wedged and free hepatic venous pressures. Adequate occlusion was confirmed by injection of 5 mL of iodinated radiologic contrast medium. Permanent tracings were recorded. Portal hypertension was defined as an HVPG ≥ 5 mmHg.

C-reactive protein and p-selectin measurement

C-reactive protein (DY1707; R&D Systems Europe, France) concentrations were measured in patients' plasma samples according to the manufacturer's instructions.
Plasma levels of P-Selectin were measured using a high-sensitivity immunoassay from Meso Scale Discovery (MSD; R-PLEX Human P-Selectin Assay, Cat No: F21ZM), according to the manufacturer's instructions. Briefly, biotinylated capture antibodies specific for P-Selectin were immobilized onto streptavidin-coated 96-well plates. Following a blocking step to reduce nonspecific binding, plasma samples diluted 1:20 in assay diluent, along with a supplied calibrator, were added to the wells and incubated to allow binding. After washing to remove unbound components, a detection antibody conjugated to a SULFO-TAG label was added. The SULFO-TAG emits light upon electrochemical stimulation in the presence of the ECL substrate provided in the MSD GOLD Read Buffer. Electrochemiluminescence (ECL) signals were generated by applying an electric current using the MESO QuickPlex SQ 120 instrument. The

resulting ECL intensity, proportional to the amount of P-Selectin in the sample, was quantified against the standard curve.

Supplementary tables

Table S1.

Name	Outcome cohort	PN-1 liver	TGA cohort	ROTEM cohort	CLA cohort	PN-1 and PAI-1 platelet cohort
Detailed description	Table 1	Suppl Table 4	Table 1	Table 1	Suppl Table 4	Suppl Table 4
Sample	PFP (peripheral vein)	PFP (hepatic vein and superior vena cava)	PRP and PFP		PFP	Washed platelets and supernatant
Aim	To measure plasma PN-1 concentration	To determine if the liver is the source of plasma PN-1 in cirrhosis	To assess platelet and plasma PN-1 role in coagulation	To assess platelet and PN-1 role in fibrinolysis	To assess the role of plasma PN-1 in fibrinolysis	To assess PN-1 / PAI-1 expression in platelet and PN-1 / PAI-1 released by activated platelet
Population	212 patients with advanced chronic liver disease all severity stages	24 patients with cirrhosis all severity stages	10 patients with cirrhosis Child-Pugh B/C stable		10 patients with cirrhosis and known elevated plasma PN-1 concentration	7 patients with cirrhosis Child-Pugh B/C stable
Control group	30 healthy individuals	-	10 healthy individuals*		-	7 healthy individuals

Abbreviations: CLA: clot lysis assay; PFP: platelet free plasma; PRP: platelet rich plasma

Table S2. Plasma PN-1 concentration according to clinical characteristics of patients in the outcome cohort

		n	Plasma PN-1 concentration (ng/mL)	p value
Gender				
- Male		212	0.12 (0-1.01)	**0.04**
- Female			0.49 (0-1.84)	
Cardiovascular risk factors	Arterial hypertension	212		
	- No		0.39 (0-1.61)	**0.04**
	- Yes		0 (0-0.94)	
	Smoking			
	- No		0.08 (0-1.25)	0.20
	- Yes		0.49 (0-1.65)	
	Diabetes			
	- No		0.46 (0-1.62)	**0.001**
	- Yes		0 (0-0.57)	
	Dyslipidemia			
	- No		0.29 (0-1.39)	0.20
	- Yes		0 (0-0.96)	
Causes of liver disease	Excessive alcohol consumption	212		
	- No		0.04 (0-1.01)	**0.04**
	- Yes		0.42 (0-1.59)	
	MASLD			
	- No		0.32 (0-1.52)	**0.01**
	- Yes		0 (0-0.83)	
	Hepatitis C			
	- No		0.29 (0-1.33)	0.39
	- Yes		0.12 (0-1.50)	
	Hepatitis B			
	- No		0.19 (0-1.45)	0.74
	- Yes		0.38 (0-1.27)	
	Other			
	- No		0.19 (0-1.33)	0.39
	- Yes		0.47 (0-2.19)	
Ascites				
- Absence		212	0 (0-1.14)	**0.02**
- Presence			0.41 (0-1.66)	
Large varices esophageal or history of band ligation		137		
- Absence			0.35 (0-1.41)	0.90
- Presence			0.38 (0-1.40)	
Hepatocellular carcinoma				
- Absence		212	0.39 (0-1.79)	**0.007**
- Presence			0 (0-0.70)	
Beta blocker treatment				
- No		211	0.26 (0-1.42)	0.27
- Yes			0.04 (0-1.15)	

Data are expressed as median (range) and were compared using the Mann-Whitney test.
Abbreviation: MASLD: metabolic dysfunction-associated steatotic liver disease

Table S3. Correlations of plasma PN-1 concentration with clinical, laboratory and hemodynamic features in the outcome cohort

	n	Correlation coefficient	p
Age (years)	212	-0.307	**< 0.0001**
Body Mass Index (kg/m^2)	212	-0.034	0.62
Model for end-stage liver disease (MELD)	212	0.283	**< 0.0001**
Serum sodium (mmol/L)	212	-0.160	**0.02**
Serum creatinine (µmol/L)	212	-0.157	**0.02**
Serum AST (ULN)	212	0.242	**< 0.001**
Serum ALT (ULN)	211	0.74	0.29
Serum bilirubin (µmol/L)	212	0.341	**< 0.0001**
Serum albumin (g/L)	212	-0.282	**< 0.0001**
Leukocytes (10^9/L)	212	0.051	0.46
Hemoglobin (g/dL)	212	-0.207	**0.002**
Platelet count (10^9/L)	212	-0.137	0.05
C Reactive protein (mg/L)	186	0.293	**< 0.0001**
Prothrombin rate (%)	212	-0.353	**< 0.0001**
HVPG (mmHg)	200	0.250	**< 0.001**
Heart rate (bpm)	208	0.006	0.93
Mean arterial pressure (mmHg)	208	-0.178	**0.01**
Right atrial pressure (mmHg)	203	0.054	0.44
Mean pulmonary artery pressure (mmHg)	193	-0.36	0.62
Cardiac index (L/min/m2)	192	0.208	**0.004**

Association between plasma PN-1 concentration and clinical, laboratory or hemodynamic features were investigated with Spearman's correlation analyses.

Abbreviation: ALT: alanine aminotransferase; AST: aspartate aminotransferase; bpm: beat per minute; HVPG: hepatic venous pressure gradient

Table S4. Characteristics of the PN-1 liver and PN-1 platelet and CLA cohort

	n	PN-1 liver (n = 24)	n	PN-1 and PAI-1 platelet (n = 7)	n	CLA cohort (n = 10)
**Clinical features**						
Age (years)	24	58 (47-64)	7	52 (45-57)	10	58 (42-62)
Male gender – N (%)	24	15 (63)	7	4 (57)	10	7 (70)
Body Mass Index (kg/m²)	24	27.1 (23.6-29.6)	7	23.7 (22.4-29)	10	25.1 (18.2-30.4)
Cardiovascular risk factors – N (%)<br>- Hypertension<br>- Smoking<br>- Diabetes<br>- Dyslipidemia	 24<br>23<br>24<br>24	 8 (23)<br>8 (35)<br>8 (33)<br>3 (13)	7	 2 (29)<br>2 (29)<br>1 (14)<br>0		 3 (30)<br>3 (30)<br>1 (10)<br>0
Causes of liver disease *<br>- Excessive alcohol consumption<br>- MASLD<br>- Hepatitis C<br>- Hepatitis B<br>- Other	24	 9 (38)<br>10 (42)<br>2 (8)<br>3 (13)<br>4 (16)	7	 6 (86)<br>2 (29)<br>0<br>0<br>1 (14)		 7 (70)<br>2 (20)<br>2 (20)<br>1 (10)<br>1 (10)
Ascites**	24	8 (33)	7	3 (43)		4 (40)
Hepatocellular carcinoma	24	0	7	0		2 (20)
Child Pugh Class<br>- A<br>- B<br>- C	24	 14 (60)<br>5 (21)<br>7 (29)	7	 0<br>4 (57)<br>3 (43)		 4 (40)<br>3 (30)<br>3 (30)
Model for end-stage liver disease (MELD)	19	13 (8-16)	7	16 (15-16)	10	14 (8-17)
**Laboratory data**						
Serum sodium (mmol/L)	24	138 (134-139)	7	136 (134-139)	10	135 (135-139)
Serum creatinine (µmol/L)	24	63 (51-81)	7	51 (49-60)	10	62 (56-73)
Serum AST (ULN)	15	56 (42-64)	7	59 (38-69)	10	49 (39-81)
Serum ALT (ULN)	15	25 (21-42)	7	28 (20-31)	10	32 (19-41)
Serum bilirubin (µmol/L)	23	32 (14-44)	7	40 (28-60)	10	45 (12-84)
Serum albumin (g/L)	15	32 (29-38)	7	32 (29-36)	10	30 (26-39)
Leukocytes (10^9/L)	24	5.2 (4-8)	7	5.8 (3.4-6.6)	10	4.9 (4.1-6.9)
Hemoglobin (g/dL)	24	11.9 (9.7-13.3)	7	11 (10.3-13)	10	12.8 (11.5-13.6)
Platelet count (10^9/L)	24	95 (66-143)	7	92 (67-98)	10	129 (98-171)
C Reactive protein (mg/L)	10	5.5 (3.5-12.1)	7	3 (2-10)	10	10 (6-45)
Prothrombin rate (%)	16	66 (50-71)	7	44 (39-54)	10	63 (55-77)
**Hemodynamic data**						
HVPG (mmHg)	24	16 (11-24)	6	18 (16-24)	9	15 (9-18)
Heart rate (bpm)	24	75 (65-88)	6	73 (65-87)	9	73 (66-90)
Mean arterial pressure (mmHg)	24	93 (80-106)	6	94 (66-100)	9	90 (80-97)
Right atrial pressure (mmHg)	24	6 (3-8)	6	5 (3-9)	9	6 (4-8)
Mean pulmonary artery pressure (mm Hg)	24	15 (11-20)	6	16 (13-20)	9	17 (14-22)

Cardiac index (L/min/m2)	24	3.6 (3-5)	6	4.1 (3.9-5.9)	9	3.2 (2.7-5)
Beta blocker treatment	24	8 (33)	7	3 (43)		4 (40)

Data are expressed as median (range) or absolute value (percentage).

* Patients can have several causes of liver disease associated

** Ascites presence was defined here as the presence of mild/moderate or abundant ascites

(referring to 2 or 3 points on the Child-Pugh classification)

Abbreviation: ALT: alanine aminotransferase; AST: aspartate aminotransferase; BPM: beat per minute; MASLD: metabolic dysfunction-associated steatotic liver disease; ULN: upper limit of normal

Table S5. Results of rotational thromboelastometry in PRP in healthy individuals and patients with cirrhosis adjusted to blood count.

PRP	Healthy individual (n = 5)		Patient with cirrhosis (n = 10)	
	Irrelevant IgG	Anti-PN-1	Irrelevant IgG	Anti-PN-1
**Without tPA**				
Clotting time (sec)	56 (44.5-61.5)	60 (52-62)	65 (58-69)#	66 (62-70)#
Clot formation time (sec)	49 (46.5-52)	52 (45.5-53.5)	62 (52-92)#	71 (56-107)** ##
Alpha angle (°)	81 (80-82)	81 (80.5-82.5)	79 (76-80) #	78 (75-79)* ##
Maximum clot firmness (mm)	65 (63.5-66.5)	57 (54.5-61)	52 (44-57) ###	42 (34-46)** ###
Amplitude at 30 minutes (mm)	64 (61.5-65.5)	53 (48.5-57.5)	49 (41-54) ###	32 (26-34)** ###
Lysis onset time (min)[1]	3353 (2882-3387)	2570 (2188-2769)	2497 (2061-3081)	835 (722-1398)** ###
Lysis index 30 minutes (%)	97 (96.5-98.5)	93 (89-94.5)	94 (88-96) ##	75 (72-79)** ###
Lysis index 45 minutes (%)	90 (86.5-91)	84 (80-85.5)	85 (80-90)	72 (66-74)** ###
Maximum lysis (%)	17 (16.5-21)	22 (21.5-24.5)	25 (18-26)	31 (28-37)** ##
**With tPA**				
Clotting time with tPA (sec)	55 (49-61)	58 (51-61)	65 (60-70)#	68 (59-74)#
Clot formation time with tPA (sec)	47 (46.5-51)	51 (45-53)	65 (51-92)#	70 (63-119)** ##
Alpha angle with tPA (°)	81 (80.5-82)	81 (80-82)	78 (77-80) #	78 (74-80)* #
Maximum clot firmness with tPA (mm)	65 (63.5-66.5)	60 (57-60)	53 (45-57) ###	41 (32-44)** ###
Amplitude at 30 minutes with tPA (mm)	63 (61-65)	53 (51.5-56)	45 (34-50) ###	21 (5-29) ###
Lysis onset time with tPA (min)	2777 (2508-2978)	2305 (2077-2457)	1927 (1691-2094) ###	921 (729-1172)** ###
Lysis index 30 minutes with tPA (%)	97 (96-98)	92 (88.5-93.5)	87 (74-94) ###	69 (12-72)** ###
Lysis index 45 minutes with tPA (%)	87 (80.5-88.5)	78 (67.5-82)	1 (0-47) ###	1 (0-5) ##
Lysis time with tPA (sec)[2]	3465	3102	2432 (2143-2955) ##	2362 (1815-2623)** ##

Maximum lysis with tPA (%)	71 (37.5-91.5)	87 (49-94)	100 (100-100) ###	100 (100-100) ##

Data are expressed as median (range).

* indicates the statistical significance for the comparisons between the condition with irrelevant IgG and anti-PN-1 antibody (in the group patients with cirrhosis or in the group of healthy individuals). Comparisons were performed using the Wilcoxon test. * $p < 0.05$ for PN-1 *vs.* PRP irrelevant IgG, ** $p < 0.01$, *** $p < 0.001$

# indicates the statistical significance for the comparisons between patients with cirrhosis and healthy individuals (irrelevant IgG in healthy individuals *vs.* irrelevant IgG in patients with cirrhosis, or anti-PN-1 in healthy individuals *vs.* anti-PN-1 in patients with cirrhosis). Comparisons were performed using the Mann Whitney test. $^{\#}$ $p < 0.05$ for patients with cirrhosis irrelevant IgG or anti-PN-1 *vs.* healthy individuals with irrelevant IgG or anti-PN-1, $^{\#\#}$ $p < 0.01$ $^{\#\#\#}$ $p < 0.001$

1. In the condition without tPA, LOT occurred in 5 healthy individuals and 9 patients with cirrhosis
2. In the condition with tPA, LT occurred in 1 healthy individual and 10 patients with cirrhosis

Supplementary figures

Fig. S1. Comparison of the effect of PN-1 inhibition on thrombin generation assay parameters between patients with cirrhosis and healthy individuals on platelet rich plasma

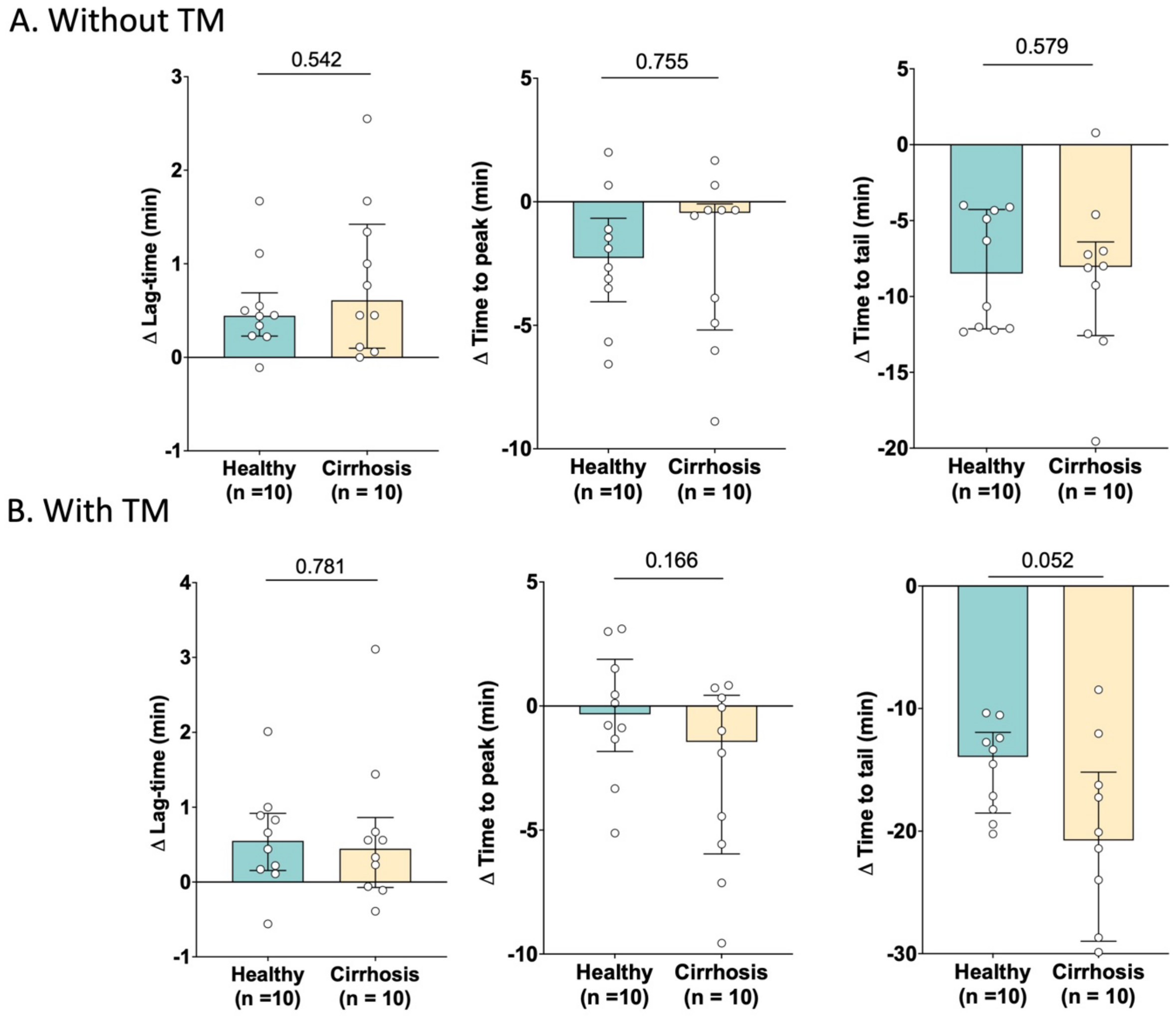

(A) Changes in lag-time, time to peak and time to tail values with anti-PN-1 antibody *vs.* irrelevant IgG without thrombomodulin (B) Changes in lag-time, time to peak and time to tail values with anti-PN-1 antibody *vs.* irrelevant IgG with thrombomodulin

The upper end of the box corresponds to the median and horizontal bars indicate the interquartile. Comparisons were made using the Mann Whitney test.

Abbreviation: IgG: immunoglobulin g; PN-1: protease nexin 1; TM: thrombomodulin

Fig. S2. Comparison of the effect of PN-1 inhibition on thrombin generation assay parameters between patients with cirrhosis and healthy individuals on platelet poor plasma

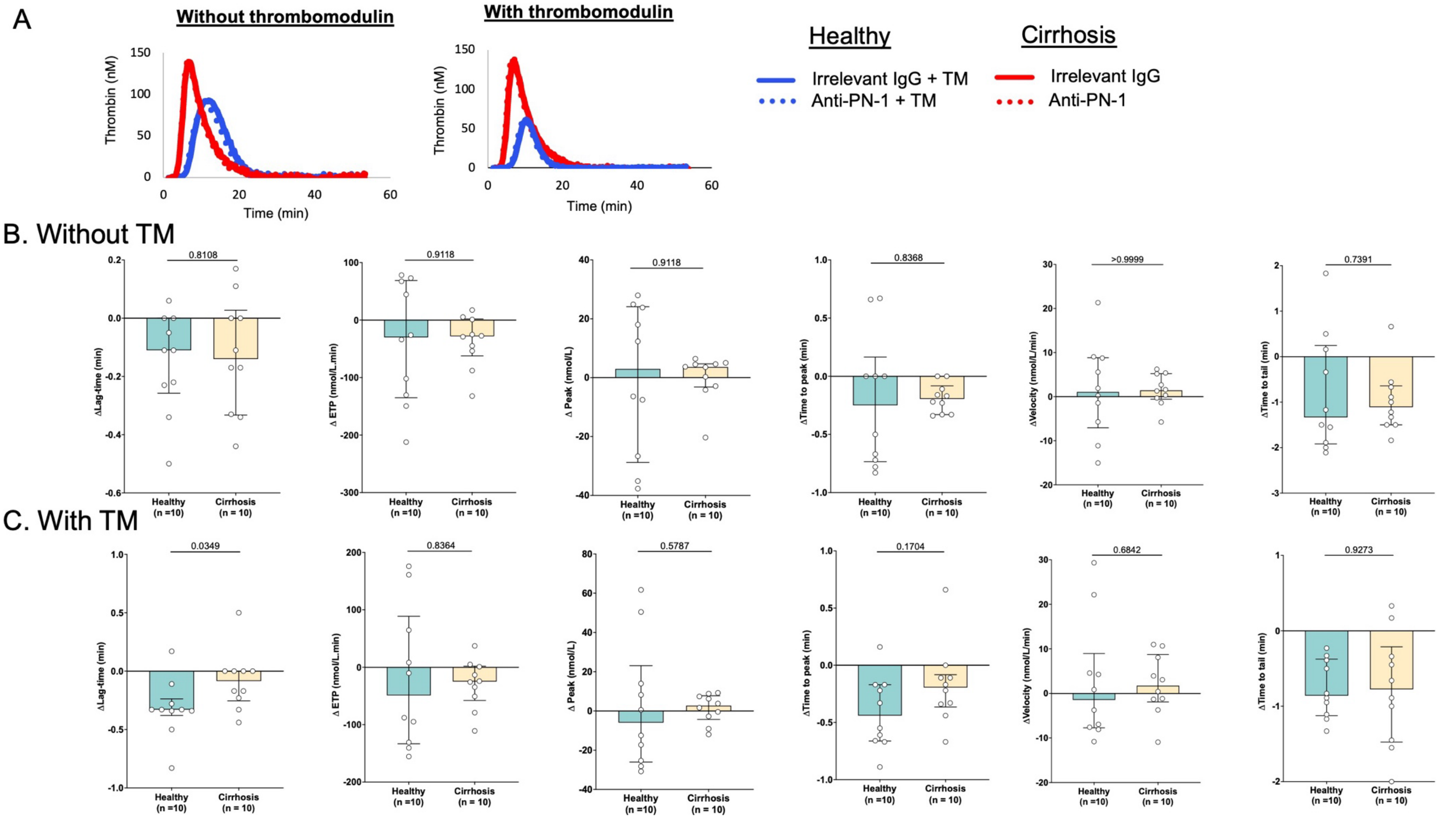

(A) Changes in lag-time, peak, ETP, time to peak, velocity index and time to tail values with anti-PN-1 antibody *vs.* irrelevant IgG without thrombomodulin (B) Changes in lag-time, peak, ETP, time to peak, velocity index and time to tail values with anti-PN-1 antibody *vs*. irrelevant IgG without thrombomodulin values with anti-PN-1 antibody *vs.* irrelevant IgG with thrombomodulin

The upper end of the box corresponds to the median and horizontal bars indicate the interquartile. Comparisons were made using the Mann Whitney test.

Abbreviation: ETP: endogenous thrombin potential; IgG: immunoglobulin g; PN-1: protease nexin 1; TM: thrombomodulin

Fig. S3. **Effect of PN-1 inhibition on clot lysis assay in PFP from patients with cirrhosis with elevated plasma PN-1 concentration**

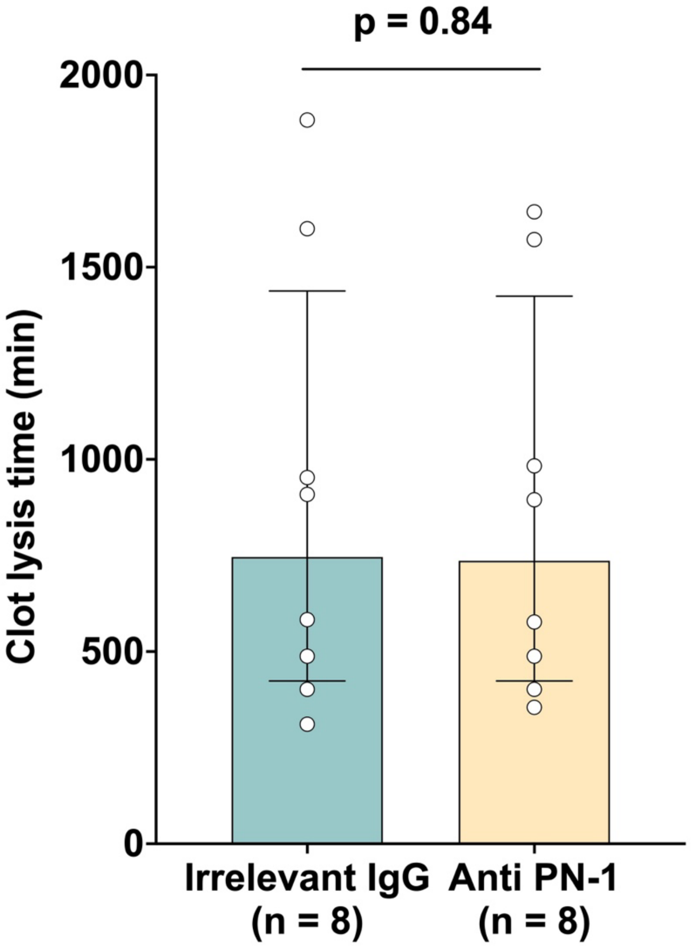

Clot lysis time measured in only 8 patients out of the 11 tested because significant fibrinolysis did not occur in 3 patients.

Fig. S4. Comparison of the effect of PN-1 inhibition on rotational thromboelastometry parameters between patients with cirrhosis and healthy individuals on platelet rich plasma with platelet count adjusted to patients or healthy individuals blood count

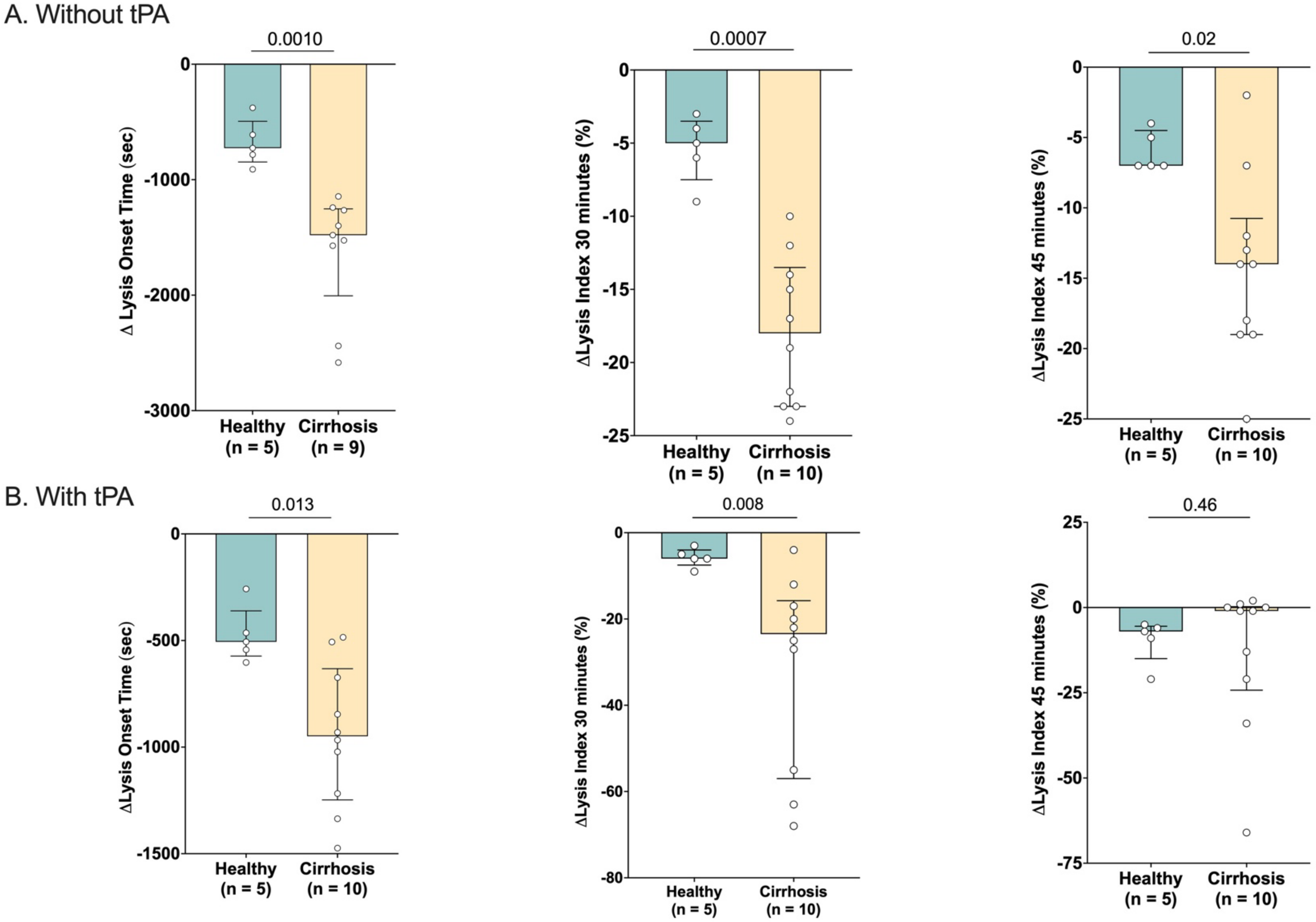

(A) and (B) Changes in Lysis Onset Time, Lysis Index 30, Lysis Index 45 values with anti-PN-1 antibody *vs.* irrelevant IgG without tPA (A) and with tPA (B)

In the condition without tPA, LOT occurred in 5 healthy individuals and 9 patients with cirrhosis.
Comparisons were made using the Mann-Whitney test.
Abbreviation: IgG: immunoglobulin g; PN-1: protease nexin 1; tPA: tissue plasminogen activator

Fig. S5. PA-1 concentration in the supernatant after platelet activation (TRAP-6) or platelet lysis (nitrogen)

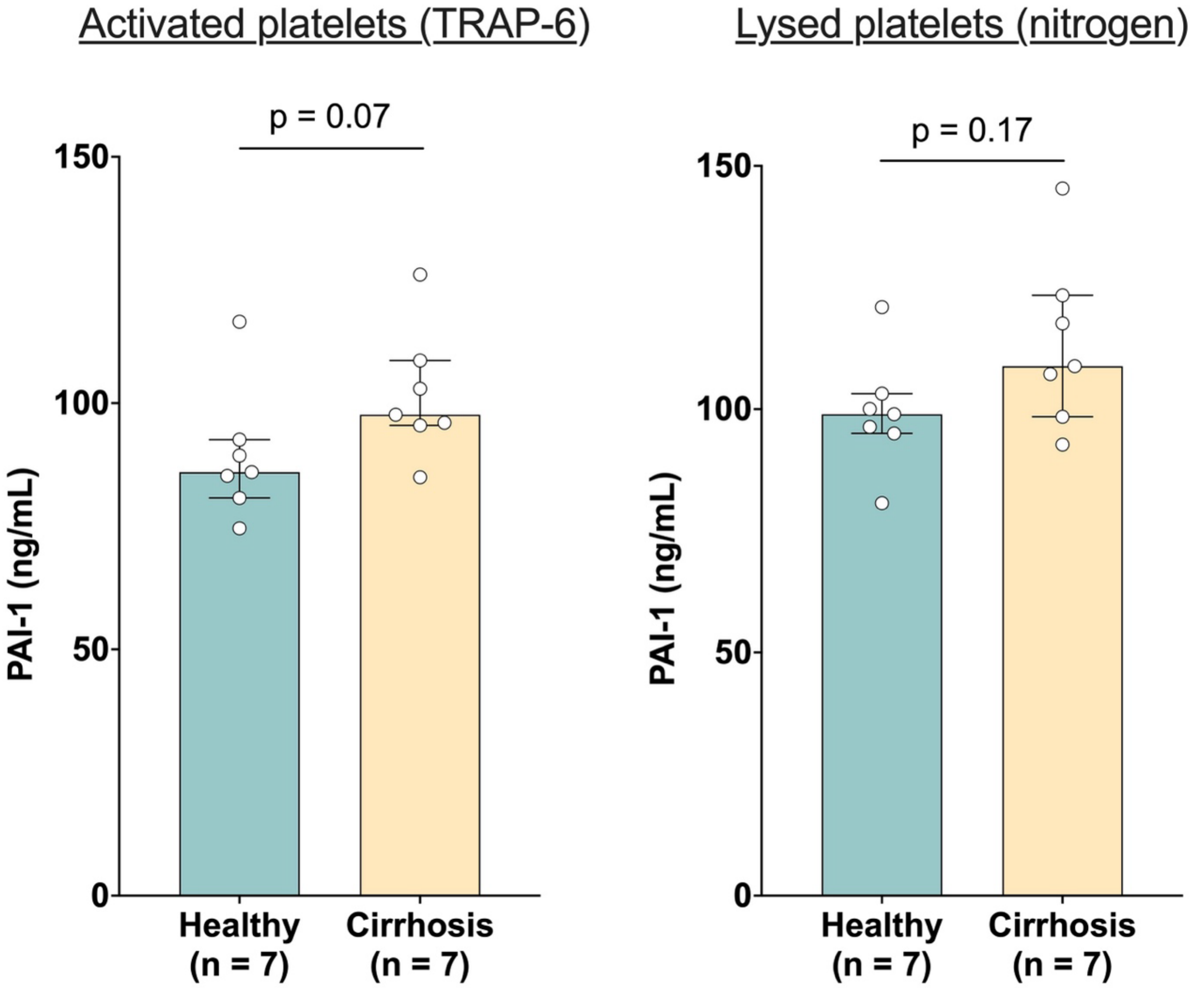

PAI-1 concentration in the supernatant of washed platelet after (A) platelet activation (TRAP-6); (B) platelet lysis (nitrogen) in 7 healthy individuals and 7 patients with stable decompensated cirrhosis Child-Pugh B/C

Comparisons were made using the Mann-Whitney test.

Abbreviation: PAI-1: plasminogen activator inhibitor type 1, TRAP-6: thrombin receptor activated platelet-6
