# Supplementary material for: Platelet protease nexin-1 limits fibrinolysis in patients with cirrhosis
Source: JHEP Rep. 2025 Aug 26;7(12):101563. doi: 10.1016/j.jhepr.2025.101563 (PMC12639314; doi:10.1016/j.jhepr.2025.101563)
Supplement: Multimedia component 2 [file mmc2.docx]

**JHEP Reports**

**CTAT methods**

Tables for a “Complete, Transparent, Accurate and Timely account” (CTAT) are now mandatory for all revised submissions. The aim is to enhance the reproducibility of methods.

- Only include the parts relevant to your study
- Refer to the CTAT in the main text as ‘Supplementary CTAT Table’
- Do not add subheadings
- Add as many rows as needed to include all information
- Only include one item per row

**If the CTAT form is not relevant to your study, please outline the reasons why:**

|  |
| --- |

- 1. **Antibodies**

| **Name** | **Citation** | **Supplier** | **Cat no.** | **Clone no.** |
| --- | --- | --- | --- | --- |
| Blocking PN-1 antibody | Aymonnier K, Kawecki C, Venisse L, et al. Targeting protease nexin-1, a natural anticoagulant serpin, to control bleeding and improve hemostasis in hemophilia. Blood 2019;134:1632-1644. | Homemade Antibody |  | Ab n°2 |
| Irrelevant IgG | Aymonnier K, Kawecki C, Venisse L, et al. Targeting protease nexin-1, a natural anticoagulant serpin, to control bleeding and improve hemostasis in hemophilia. Blood 2019;134:1632-1644. | Jackson | 011-000-003 |  |

- 1. **Cell lines**

| **Name** | **Citation** | **Supplier** | **Cat no.** | **Passage no.** | **Authentication test method** |
| --- | --- | --- | --- | --- | --- |
|  |  |  |  |  |  |

- 1. **Organisms**

| **Name** | **Citation** | **Supplier** | **Strain** | **Sex** | **Age** | **Overall n number** |
| --- | --- | --- | --- | --- | --- | --- |
|  |  |  |  |  |  |  |

- 1. **Sequence based reagents**

| **Name** | **Sequence** | **Supplier** |
| --- | --- | --- |
|  |  |  |

- 1. **Biological samples**

| **Description** | **Source** | **Identifier** |
| --- | --- | --- |
| Platelet free plasma | Human |  |
| Platelet rich plasma | Human |  |

- 1. **Deposited data**

| **Name of repository** | **Identifier** | **Link** |
| --- | --- | --- |
|  |  |  |

- 1. **Software**

| **Software name** | **Manufacturer** | **Version** |
| --- | --- | --- |
| Prism | GraphPad | 10 |
| SPSS | IBM | 29 |

- 1. **Other (e.g. drugs, proteins, vectors etc.)**

| **Name** | **Source** | **Supplier** |
| --- | --- | --- |
| Thrombomodulin | Source : Rabbit | Cryopep, reference, 9-RABTMM-4202) |

- 1. **Please provide the details of the corresponding methods author for the manuscript:**

| Prof. Pierre-Emmanuel Rautou  Service d’hépatologie  Hôpital Beaujon  100. boulevard du Général Leclerc  92110 Clichy, France  Tel: +33.1.40.87.52.83  Fax: +33.1.40.87.55.30  pierre-emmanuel.rautou@inserm.fr |
| --- |

**2.0 Please confirm for randomised controlled trials all versions of the clinical protocol are included in the submission. These will be published online as supplementary information.**

|  |
| --- |
